# Artificial Intelligence in Orthodontics: From Laboratory Benchmarks to Clinical Care

**DOI:** 10.7759/cureus.111151

**Published:** 2026-06-19

**Authors:** Omar H Alkadhi

**Affiliations:** 1 Department of Preventive Dentistry, Riyadh Elm University, Riyadh, SAU

**Keywords:** artificial intelligence, cbct segmentation, cephalometry, clinical decision-making, deep learning, large language models, machine learning, orthodontics

## Abstract

Artificial intelligence (AI) is now used in routine orthodontic practice, not only in research, but how well it works depends heavily on the application. This narrative review synthesises peer-reviewed studies published between 2019 and early 2026 across seven task domains: cephalometric landmark detection, cervical vertebral maturation (CVM) staging, treatment-decision support for extraction and orthognathic surgery, cone-beam computed tomography (CBCT) segmentation, aligner monitoring, treatment-outcome prediction, and large language model (LLM) patient communication, which differ markedly in their level of maturity. In cephalometric landmark detection, mean radial errors ran from roughly 1.0 mm in three dimensions to about 1.37 mm in two dimensions, and pooled detection reached about 81% at the 2 mm threshold. CBCT segmentation reached pooled Dice similarity coefficients of 0.93 for teeth and 0.91 for the maxilla on multicentre data. Extraction-decision models performed with a sensitivity of 0.70 and a specificity of 0.90, yet lost as much as 20% of their accuracy once tested across institutions. Remote aligner monitoring reduced in-office attendances by roughly 1.68 to 3.5 visits over a treatment course, although the same platforms detected periodontal status poorly, with sensitivities of only 0.53, 0.35, and 0.22 for plaque and calculus, gingivitis, and recession, respectively. Soft-tissue prediction after orthognathic surgery remained inaccurate at the lip and chin. Patients tended to prefer LLM-generated information about their treatment, whereas orthodontic experts rated the same material less favourably. Three problems recurred across every domain examined: training data confined to single centres, narrow demographic representation, and an absence of independent external validation. None of these applications removed interpretive responsibility from the clinician, who retained the analytical decision, even where AI reduced the computational burden.

## Introduction and background

Today’s orthodontic records routinely comprise intraoral scans, panoramic radiographs, lateral cephalograms, and, far more commonly than five years ago, cone-beam computed tomography (CBCT) volumes. All of it used to be measured by hand; one clinician did the measuring, and that clinician's eye was the only quality check. This has changed. Software now traces cephalograms on its own, segments CBCT volumes without supervision, and follows aligner cases between appointments through a patient’s smartphone, though how widely a practice has adopted any of this and how rigorously a given product has been validated vary from one office to the next. Artificial intelligence (AI) has reached furthest into clinical work in seven areas: cephalometric analysis, skeletal maturation staging, extraction and orthognathic surgery planning, three-dimensional segmentation, aligner monitoring, treatment outcome prediction, and patient communication. These are the domains that are reviewed here.

The review literature has grown alongside the technology, and much of it repeats. Scoping and systematic reviews now cover almost every application, often over the same ground [1-6]. The underlying primary studies are a mixed set, from single-centre diagnostic-accuracy reports to randomised trials and meta-analyses, and have to be weighed accordingly. Nordblom et al. [7] flagged three recurring problems: most training data come from one centre, external generalisability is rarely tested, and few products hold regulatory clearance. The FDI issued its own communiqué on AI in dentistry in 2025 [8].

Most of these reviews ask broad questions. The clinically useful question is narrower: which tools are accurate enough to use and which are not? That is what is addressed, across the seven domains above, chosen for what they mean in everyday practice rather than for how much has been published on them. Specifically, a domain was considered clinically relevant if it represented a decision or measurement step that an orthodontist performs routinely in patient assessment or treatment planning and for which AI tools are commercially available or in active clinical evaluation.

## Review

Materials and methods

The review followed the Scale for the Assessment of Narrative Review Articles (SANRA) reporting items [9]. The seven domains were set in advance (cephalometric landmark detection, cervical vertebral maturation (CVM) staging, extraction and orthognathic surgery decision support, CBCT segmentation, aligner monitoring, treatment-outcome prediction, and large language model (LLM) patient communication). These domains were chosen because the relevant AI tools are either on the market or in active clinical study, and because orthodontists encounter all seven in routine work. PubMed/Medical Literature Analysis and Retrieval System Online (MEDLINE), Scopus, Web of Science, IEEE Xplore, and the Consensus literature engine were searched for English-language papers published between January 2019 and March 2026 (2019 was chosen as the start year because it marks the emergence of deep learning as the dominant methodological paradigm in orthodontic AI, evident in the first published applications in each domain reviewed here), pairing AI terms (“artificial intelligence”, “deep learning”, “machine learning”, “convolutional neural network”) with descriptors for each domain. No original quantitative synthesis was performed; all pooled estimates, including sensitivity, specificity, Dice coefficients, mean radial errors, and heterogeneity statistics, were taken directly from previously published systematic reviews and meta-analyses and are reported here in the form given by those source studies.

Eligibility criteria were kept deliberately broad in order to capture the full spread of evidence, encompassing diagnostic-accuracy studies, prospective and retrospective clinical evaluations, randomised controlled trials, systematic reviews, and meta-analyses addressing any of the seven domains. No formal PRISMA screening was applied, consistent with the narrative design, and inclusion was guided by clinical relevance. Non-English papers, conference abstracts not subsequently published as peer-reviewed articles, editorials, and preprints were excluded. Where a domain had been covered by more than one synthesis, the most recent synthesis carrying a structured risk-of-bias appraisal, typically a meta-analysis employing Quality Assessment of Diagnostic Accuracy Studies-2 (QUADAS-2), was preferred. Given the wide range of study designs and weights, from a single hospital’s diagnostic-accuracy series to a multicentre meta-analysis, the evidence is reported narratively rather than pooled.

Each metric is reported as given in the source study. Diagnostic-accuracy studies provided sensitivity, specificity, and the area under the receiver operating characteristic curve (AUC); segmentation studies provided the Dice similarity coefficient, with intersection-over-union where reported; and landmarking studies provided mean radial error together with the rate of successful detection at clinically meaningful thresholds. Risk of bias was not scored at the level of the primary studies, for which the cited meta-analyses should be consulted.

Cephalometric landmark detection

By volume of published accuracy data and breadth of architectural approaches tested, two-dimensional cephalometric landmark detection was the most extensively studied of the seven domains, though external validation across demographically diverse populations and clinical impact data remained variable. Working from 2,075 lateral cephalograms drawn from two institutions, Kim et al. [10] trained a stacked-hourglass network on 23 landmarks and brought the mean point-to-point error to 1.37 ± 1.79 mm. Lee et al. [11] took the same problem in a different direction, using Bayesian convolutional neural networks (CNNs) that attach an explicit confidence region to every prediction; their approach roughly halved the error at Gonion, long the largest single source of error in automated cephalometry. A YOLOv3-based system tested by Bulatova et al. [12] showed no statistically significant difference from a calibrated examiner's manual tracing for 12 of the 16 landmarks examined. The largest of these studies, from Kim et al. [13], trained cascade networks on 3,150 cephalograms gathered across 10 Korean university hospitals and reached 1.36 ± 0.98 mm overall, against an inter-examiner variability of 1.31 ± 1.13 mm; for practical purposes, the model and the clinicians were indistinguishable. Even so, hospital, sensor type, and machine model each affected accuracy.

Performance in three dimensions has proved more variable. On a presurgical CT sample, Dot et al. [14] reported a mean localisation error of 1.0 ± 1.3 mm and a detection rate of 95.4% within 3 mm. Sahlsten et al. [15] ran a model across a mixed dataset of 309 Finnish and Thai CBCT scans; 33 of the 46 landmarks, and every cephalometric measurement derived from them, came out with no significant difference between the two cohorts. Liu et al. [16] built a multicentre model that held mean radial error below 1.3 mm on both spiral CT and CBCT, and found that junior specialists landmarked 28.9% more accurately when they worked alongside it. Direct numerical comparison between two-dimensional and three-dimensional accuracy figures is not valid, as the two bodies of work differ in landmark sets, distance metrics, imaging modalities, and clinical populations; what follows reflects relative research maturity within each modality rather than head-to-head performance.

Pooling 21 studies, Londono et al. [17] reported detection rates of 65%, 81%, 86%, 91%, and 96% at thresholds of 1, 2, 2.5, 3, and 4 mm, respectively. Zaborowicz et al. [18] reviewed 20 studies and judged the accuracy clinically acceptable while finding the demographic range of the training data too narrow. Pairing a beginner with an AI system raised detection at 2 mm by 5.33% over unaided reading [19]. On the available evidence, this domain was the closest of the seven to routine clinical use.

CVM staging

CVM staging assigns a growing patient to one of six stages, CS1 to CS6, read from the morphology of the C2 to C4 vertebral bodies, and is consequential for the timing of growth modification, more so now that hand-wrist radiography, the conventional alternative, is being abandoned on radiation-dose grounds. Makaremi et al. [20] first showed that a CNN could stage CVM from a lateral cephalogram. Mohammad-Rahimi et al. [21] pushed the idea further, reaching 61.6% accuracy on the full six-class problem and 82.8% on the easier three-class growth-spurt version.

Sadeghi et al. [22] pooled 25 studies and broke the results down stage by stage. Performance peaked at CS1, with a sensitivity of 0.87, a specificity of 0.97, and a diagnostic odds ratio of 213, then sagged to a sensitivity of 0.64 at the middle stage, CS3. A systematic review by Kazimierczak et al. [23] covering 18 studies put accuracy anywhere from 57% to 95%, depending on the method. Guo et al. [24] added dental maturation, sex, and chronological age to the vertebral data and reported an AUC of 0.924 for skeletal maturity.

Accuracy across these studies is, therefore, uneven, peaking at the extremes of the maturation sequence (CS1 before the growth spurt and CS6 after it, as quantified by Sadeghi et al. [22]) and falling at the intermediate stages, precisely where the timing of growth modification is most dependent on the staging result.

Treatment-decision support: extraction

The extraction decision is one of the weightiest in treatment planning, and several models tackle it head-on. Li et al. [25] trained a neural network on cephalometric and clinical variables and reached 94% accuracy, with crowding, ANB angle, and curve of Spee doing most of the predictive work. Then the data widened, and the figure dropped. Across a multi-provider United States sample of 838 patients, Etemad et al. [26] got 75% to 79% on the same yes-or-no decision, below the earlier single-centre figures and consistent with a model overfitted to the case mix of one institution.

The meta-analysis by Ziaei et al. [27] gathered seven studies and 6,261 patients, pooling to a sensitivity of 0.70 (95% CI 0.61 to 0.78) and a specificity of 0.90 (95% CI 0.87 to 0.92). Heterogeneity was high, though, with I² at 96.7% for sensitivity and 93.7% for specificity. An evidence-based dentistry commentary summarising the Ziaei et al. [27] findings traced the best results to CNN architectures, the ResNet and VGG variants in particular [28]. Mason et al. [29] tried four models on a racially and ethnically diverse sample of 393 patients; logistic regression, the support vector machine, and the neural network all returned AUCs between 0.91 and 0.93. The generalisability problem showed itself plainly when Etemad et al. [30] trained on one university’s data and tested on another’s, and accuracy fell by as much as 20%.

Ryu et al. [31] changed the input altogether, feeding the model intraoral photographs instead of cephalometric measurements, and reached an AUC of 0.961 for the maxillary extraction decision. Even so, the cross-institutional drop that Etemad et al. [30] documented, a model trained at one centre losing a good part of its accuracy on patients from another, is still what most limits this domain.

Treatment-decision support: orthognathic surgery

The decision between surgical correction and orthodontic camouflage in skeletal malocclusion has generated its own line of AI work, methodologically similar to the extraction models but carrying higher clinical stakes and concentrated in a small number of countries. In 316 patients, Choi et al. [32] reported 96% accuracy for the straight surgery-or-not call and 91% when the model also had to name the surgery type and extraction pattern. Jeong et al. [33] showed that a facial photograph on its own can flag a profile needing surgery, at 89.3% accuracy. Testing 10 models on 920 patients, 558 Class II and 362 Class III, de Oliveira et al. [34] saw test-set accuracy between 0.70 and 0.82, strongest in the Class III cases.

Notwithstanding the methodological overlap with the extraction work, this domain has undergone considerably less external testing, and no independent group has reproduced the Choi et al. [32] figures in a non-Korean population.

CBCT segmentation

Several of the remaining tasks are contingent on prior segmentation of teeth and bone from the CBCT volume. Wang et al. [35] handled jaw and teeth together in a single network, with Dice coefficients of 0.934 and 0.945. Lahoud et al. [36] reported an intersection-over-union of 0.87 for teeth and surface deviation under 10 micrometres, completing the segmentation 12 times faster than the semi-automated method it replaced. The biggest study, by Cui et al. [37], ran on 4,938 CBCT scans from 15 centres and averaged Dice scores of 0.915 for teeth and 0.930 for alveolar bone, around 500 times faster than segmenting by hand.

Pooling 30 studies, Alahmari et al. [38] reported Dice coefficients of 0.94 for the mandible, 0.93 for teeth, and 0.91 for the maxilla, which, alongside two-dimensional cephalometric landmarking, makes this the second domain accurate enough for routine clinical use.

Aligner monitoring

Remote monitoring of aligner treatment is supported by prospective evidence. Reviewing 11 studies, Sangalli et al. [39] found that it trims in-office visits by 1.68 to 3.5 per patient. Torres et al. [40] backed this up with a meta-analysis of four aligner studies (mean difference −2.75 visits, 95% CI −3.95 to −1.55) and saw a shorter time to first refinement as well, although they rated the certainty of the evidence very low.

The performance of these platforms in assessing periodontal status remains inadequate. Across 232 examinations, Snider et al. [41] tested the Dental Monitoring oral-hygiene algorithm (Dental Monitoring SAS, Paris, France). Sensitivities were low: 0.53 for plaque and calculus, 0.35 for gingivitis, and 0.22 for recession, even as specificity stayed high at 0.94, 0.96, and 0.99. Detection of tooth movement is a separate matter, on which Homsi et al. [42] showed that the platform’s three-dimensional models follow tooth movement about as accurately as iTero scans (Align Technology, Tempe, AZ, USA) over an average of 13.4 months.

Kazimierczak et al. [43] checked Diagnocat’s panoramic charting (Diagnocat Inc., San Francisco, CA, USA) against expert consensus on 4,148 teeth from 147 patients. Tooth by tooth, the accuracy ran from 94.9% to 99.9%, but only 56.5% of the full-mouth reports came back error-free once judged per patient. A CBCT follow-up was worse: Diagnocat produced a skeletal classification for 5%of patients, a vertical one for 1.7%, and a Dental Angle class for 57.6%, too little to chart a case on its own [44].

Two capabilities of remote aligner monitoring are therefore established, namely the reduction of in-office visits and the three-dimensional tracking of tooth movement, whereas periodontal assessment and full-mouth charting are not yet accurate enough to be relied upon.

Treatment-outcome prediction

Among the seven domains, outcome prediction is the least developed. In 38 patients, Haouili et al. [45] reported a mean Invisalign accuracy (Align Technology, Tempe, AZ, USA) of 50% across all movements, best for buccolingual crown tipping at 56% and worst for rotation at 46%. Castroflorio et al. [46], following 79 patients, found that teeth moved about 0.4 degrees for every degree the aligner prescribed. Saif et al. [47] reported 50.3% accuracy for anterior alignment after the first set of aligners in 40 patients, with upper-incisor intrusion the hardest to predict at 23.1%. The same predictive ceiling is evident across all three of these small prospective studies.

The prediction of soft-tissue outcomes following orthognathic surgery constitutes a separate problem. Tanikawa et al. [48] reported mean errors of 0.94 mm for a surgical system and 0.69 mm for an orthodontic-only one, both acceptable across most landmarks, with the residual error concentrated regionally. Salazar et al. [49], reviewing eight studies, found the predictions still unacceptable at the lip and chin, and Almarhoumi [50] said much the same from seven studies, blaming small samples and no external validation. Kim et al. [51] put a number on the gap: by their simulation, training a model to stay under 1.5 mm of error at the lower lip would take more than 1,700 paired surgical datasets, a figure that no published dataset approaches.

LLM patient communication

The most recent of the seven domains is the application of LLMs to patient education. When Vassis et al. [52] gave patients GPT-generated material (OpenAI, San Francisco, CA, USA) about the side effects of treatment, about 80% preferred it to the standard text from a university programme; the orthodontic experts who read the same material called it neither poor nor good. Demirsoy et al. [53] ran ChatGPT-4 through questions on clear aligners, lingual orthodontics, aesthetic braces, and temporomandibular disorders, and again, the patients rated the answers higher than the orthodontists did. Makrygiannakis et al. [54] pitted four LLMs against 10 orthodontic questions and scored Microsoft Bing Chat (Microsoft Corporation, Redmond, WA, USA) the best of them.

A consistent pattern emerges across all three studies, in which patients rated the LLM output more favourably than specialists did, and no study returned a consistently positive expert verdict, indicating a role for LLMs in patient communication that is not yet matched by demonstrated conformity to a specialist standard.

Three practical considerations bear on clinical deployment of LLMs for patient communication. First, LLMs can generate factually incorrect information without indicating uncertainty, a property known as hallucination; in an orthodontic context, this could mislead patients about treatment risks, timelines, or expected outcomes. Second, patient queries submitted through consumer-facing platforms may be processed and retained by third-party providers, raising confidentiality concerns under applicable data-protection legislation. Third, no established medico-legal framework currently assigns liability for errors in AI-generated patient information, leaving the clinician as the responsible party in the absence of formal guidance. These are not arguments against use, but they underscore the need for clinician oversight of any content before it reaches patients.

A domain-by-domain summary of performance metrics, pooled estimates, and maturity ratings is presented in Table 1.

**Table 1:** Performance of artificial intelligence systems across seven orthodontic task domains. 2D = two-dimensional; 3D = three-dimensional; SDR = success detection rate; CVM = cervical vertebral maturation; CS3 = cervical stage 3; CBCT = cone-beam computed tomography; LLM = large language model; I² = I-squared statistic for heterogeneity. Dashes (—) = no pooled estimate available. Maturity labels: 'Routine clinical use' = multiple independent multicentre validations, performance at or approaching human expert level, commercially available tools with regulatory clearance in at least one jurisdiction; 'Approaching maturity' = strong multicentre accuracy data, limited external validation across diverse populations; 'Variable; reliable at extremes' = high accuracy at early and late diagnostic stages, unreliable at the central stages where clinical decisions are most consequential; 'Useful within training population' / 'Useful, with caveat of cross-institutional drop' = good within-cohort accuracy with documented degradation on cross-institutional testing, limited independent validation; 'Validated for visit reduction' = prospective evidence for that specific outcome but not the full clinical workflow; 'Not validated for periodontal status' = absence of prospective evidence for that specific function; 'Not yet clinically reliable' = conflicting results across studies or systematic underperformance in the most clinically consequential scenarios; 'Emerging' = feasibility and proof-of-concept data only, insufficient prospective clinical evidence for routine use.

Task Domain	Representative Metric	Best Published Result	Pooled Estimate	Maturity
Cephalometric landmark detection (2D)	Mean radial error; SDR at 2 mm	1.36 ± 0.98 mm [13]	81% at 2 mm [17]	Routine clinical use
Cephalometric landmark detection (3D)	Mean localisation error	1.0 ± 1.3 mm [14]	1–2 mm typical [18]	Approaching maturity
CVM staging	Sensitivity at the central stage	0.64 sensitivity at CS3 [22]	57–95% accuracy range [23]	Variable; reliable at extremes
Extraction decision	Pooled sensitivity/specificity	0.70 / 0.90 [27]	I² 96.7% / 93.7%	Useful, with the caveat of cross-institutional drop
Orthognathic surgery decision	Binary classification accuracy	96% [32]	0.70–0.82 testing accuracy [34]	Useful within the training population
CBCT segmentation	Dice coefficient	0.915 tooth / 0.930 bone [37]	0.93 teeth / 0.91 maxilla [38]	Routine clinical use
Aligner monitoring (visits)	In-office visit reduction	−2.75 visits [40]	1.7–3.5 visits [39]	Validated for visit reduction
Aligner monitoring (periodontal)	Sensitivity for plaque/gingivitis/recession	0.53 / 0.35 / 0.22 [41]	—	Not validated for periodontal status
Outcome prediction (aligner)	Mean accuracy	50.3% [47]	~50% across major movements [45,46]	Not yet clinically reliable
Outcome prediction (orthognathic soft tissue)	Mean prediction error	0.69–0.94 mm at routine landmarks [48]	Clinically unacceptable at the lip and chin [49]	Not yet clinically reliable
LLM patient communication	Patient preference / expert rating	80% patient preference; experts neutral [52]	—	Emerging

Synthesis and clinical implications

The strength of evidence underlying these domains varies substantially. Cephalometric landmark detection and CBCT segmentation are supported by the strongest foundation: multiple independent multicentre datasets and meta-analyses with formal risk-of-bias appraisal. Extraction decision support and orthognathic planning have been synthesised in meta-analyses, but documented cross-institutional accuracy losses limit confidence in the pooled figures. For aligner remote monitoring, the visit-reduction outcome rests on prospective studies, though the certainty of evidence was rated as very low by Torres et al. [40]. Treatment-outcome prediction and LLM patient communication are supported only by small prospective cohorts and require multicentre replication before routine clinical adoption can be justified.

How mature these tools are depends on the task. Cephalometric landmarking and CBCT tooth segmentation are accurate enough for everyday use, as long as the clinician checks each result rather than taking it on trust. Extraction and orthognathic planning sit in between. They do well on patients who resemble the training set and worse on those who do not, so the model works as a second opinion, not the deciding one. Soft-tissue prediction and periodontal monitoring fall short exactly where their output would matter most. Conversational AI is a different case again, because across the comparative studies, it is patients, not specialists, who rate the output well.

One imbalance runs through the training data in every domain. Korean, Chinese, and Caucasian patients make up most of it, while patients from the Gulf region, South Asia, sub-Saharan Africa, and Indigenous populations are almost entirely missing [7]. This matters beyond sample size. Craniofacial morphology differs systematically across ethnic groups: facial convexity, mandibular plane angle, and incisor inclination vary in ways that affect cephalometric landmark positions; skeletal maturation timing and vertebral morphology patterns underlying CVM staging are not uniform across populations; and differences in cortical bone density and root morphology may influence segmentation performance. Accuracy figures from predominantly East Asian or Caucasian cohorts cannot be assumed to transfer to Gulf Arab, South Asian, or sub-Saharan African patients without independent validation on representative samples. Two further practical consequences follow. An accuracy figure measured within the training cohort overstates the performance a clinician will observe in a different population, and wherever cross-institutional testing has been performed, the figure has fallen by 20% or more [30]. The independent evaluation of Diagnocat by Kazimierczak et al. [43,44], conducted on patients the developers had not used in training, represents the methodological standard the rest of the field should adopt. Until other commercial tools are subjected to comparable external scrutiny, a vendor-reported accuracy figure is best interpreted as an upper bound rather than an expected value.

Several questions merit consideration before any of these tools is introduced into practice. The first concerns the provenance of the training data, since the demographic and institutional composition of that dataset determines how far a published accuracy figure transfers to the patients in a given practice. The second concerns whether the tool has been validated on independently collected data, a test more informative than any vendor-supplied figure. The failure mode of a model is also relevant, not only its failure rate, because systematically directional errors are more readily managed than errors distributed unpredictably across cases. Regulatory clearance, while reassuring, is not equivalent to prospective independent validation. The time a tool saves does not translate automatically into clinical capacity, since a reduced analytical burden may instead reduce the attention paid to whatever the model fails to flag.

In practical terms, cephalometric tracing, CBCT segmentation, and remote aligner monitoring are the applications for which AI is best supported on the evidence to date. Extraction decisions, soft-tissue prediction in difficult surgical cases, and full-mouth CBCT charting remain decisions for the clinician, with the model treated as one input among several, and LLMs are of use in patient communication, provided a clinician reviews the generated material first.

Several research priorities emerge from this synthesis. The most pressing issue is the development of training datasets representative of underrepresented populations, including Gulf Arab, South Asian, sub-Saharan African, and Indigenous patients, without which accuracy claims will continue to overstate real-world performance outside the source cohorts. Independent multicentre external validation of commercially deployed systems, conducted by researchers without financial ties to the evaluated product, is the methodological standard the field currently lacks for most tools. Standardised performance reporting within each domain would enable meaningful cross-study comparison; current heterogeneity in metrics, landmark sets, and outcome definitions limits the interpretability of pooled analyses. Adequately powered prospective datasets for soft-tissue prediction are specifically needed: Kim et al. [51] estimated that more than 1,700 paired surgical cases are required for consistent sub-1.5 mm error at the lower lip, yet no existing system has been trained on data approaching that volume. Finally, legal and ethical frameworks governing AI-generated patient communication, including guidance on disclosure, informed consent, and liability, remain to be established.

Limitations

This review is subject to several limitations. The search was closed in early 2026, and in a rapidly moving field, additional work will already have appeared. The domains were selected for clinical relevance rather than completeness, so that several areas, among them temporomandibular joint MRI, surgical-guide design, and growth-modification appliance simulation, receive no treatment. Risk of bias was assessed at the level of the cited meta-analyses rather than the underlying primary studies, and conclusions resting solely on unappraised primary work therefore warrant additional caution. Dental Monitoring, Diagnocat, and Invisalign occupied more space than their competitors because they have more frequently been studied independently, and the thinner coverage of WebCeph, CephX, OrthoDx, Planmeca Romexis, and Angelalign carried no implication regarding their performance. Because no formal PRISMA screen was applied, two related biases cannot be excluded: publication bias, the tendency for studies reporting favourable AI performance to be published more readily than those reporting null or negative results, may cause the accuracy figures cited in this review to overestimate performance across the broader population of deployed models; and selective citation bias, arising from the author-driven identification of relevant work in a narrative design, may mean that studies were inadvertently omitted or that certain architectural approaches or geographic cohorts are overrepresented.

## Conclusions

The question of whether AI belongs in orthodontics has, at this point, been settled by adoption. Cephalometric tracing and CBCT segmentation are in working clinical use; extraction and surgical-planning aids are useful but inconsistent across centres; soft-tissue prediction and periodontal monitoring are insufficiently validated for clinical reliance; and LLMs are increasingly present in clinician-patient communication. None of these developments transfers the responsibility for interpretation and decision away from the clinician, who retains the judgement even where AI assumes part of the analysis. The field’s present requirements are external validation across multiple centres, training data representative of more than a small number of populations, and independent evaluation of the tools already in commercial use; these are the conditions that separate a favourable laboratory metric from a result on which a clinician can depend in practice.
